# Editorial: Use of bioactives for treatment of respiratory diseases

**DOI:** 10.3389/fphar.2025.1630486

**Published:** 2025-07-03

**Authors:** Alan de Aguiar Lopes, Akinori Nagato, Frank Bezerra, Samuel Santos Valenca, Sabina Antonela Antoniu

**Affiliations:** ^1^ Department of Education, Concordia University, Québec City, QC, Canada; ^2^ Department of Biophysics and Physiology, Federal University of Juiz de Fora, Juiz de Fora, Brazil; ^3^ Department of Biological Sciences, Institute of Exact and Biological Sciences, Federal University of Ouro Preto, Ouro Preto, Brazil; ^4^ Institute of Biomedical Sciences, Federal University of Rio de Janeiro, Rio de Janeiro, Brazil; ^5^ Department of Nursing, Grigore T. Popa University of Medicine and Pharmacy, Iași, Romania

**Keywords:** chronic lung diseases, respiratory diseases, bioactive molecules, treatment, respiratory pharmacology

Chronic lung diseases (CLDs) are conditions that affect the components of the respiratory system, and they are among the leading causes of mortality and morbidity worldwide. According to the 2017 Global Burden of Diseases, Injuries, and Risk Factors study (GBD), 545 million people suffer from respiratory diseases (Soriano et al., 2020). Those diseases have etiological factors ranging from communicable or infectious agents (e.g., tuberculosis and COVID-19) to non-communicable ones (e.g., asthma, chronic obstructive respiratory disease (COPD), and lung cancer) (Vos et al., 2015; Shukla et al., 2020). Current therapies for CLDs focus on treating these factors and alleviating symptoms using antibiotics and anti-inflammatory drugs (Shukla et al., 2020). Advancements in knowledge about the molecular mechanisms and biochemical pathways involved in the development of several respiratory diseases [e.g., cellular senescence (Barnes et al., 2019), Wnt/β-catenin signaling (Shi et al., 2017)] have conducted potentiated studies on possible pharmacological targets and the development of new drugs, including bioactive molecules (BMs).

Bioactive molecules have been extensively studied as a potential treatment for respiratory diseases. The use of BM as a source of drugs has been documented for more than 40 years (Newman and Cragg, 2020). In terms of CLDs, BMs have been investigated as treatments for conditions such as COVID-19 (Zhao et al., 2024), asthma (Zhou et al., 2016; Chen et al., 2017), emphysema (Games et al., 2016), pulmonary fibrosis (Wang et al., 2023), and tuberculosis (Antunes, Won-Held Rabelo and Romeiro, 2021). These studies have shown how these molecules modify the course of these diseases in the body. Taking into consideration the research on BMs and CLDs, this Research Topic covers the recent findings on the use of BM for the treatment of CLDs.

In this Research Topic, eight contributions were published, including a review, a case study, and six original research articles.

The review by Hufnagel et al. discussed the role of caffeoylquinic acids (DCQAs) in respiratory diseases. This article provided up-to-date information on the pharmacological properties of these molecules. These properties translate into their pharmacodynamics, antioxidant, and anti-inflammatory activity. This article also reviewed the mechanisms of action of DCQAs, including NF-κB and Nrf2 pathways and the reduction of oxidative stress. This review presents compounds that warrant further investigation as antitussive agents.

The case study by Wang et al. investigated the relationship between the protective effect of aspirin against community-acquired pneumonia (CAP) in patients requiring an ICU stay. By analyzing the Medical Information Mart for Intensive Care IV (MIMIC-IV) database, the authors observed a relationship between aspirin use and reduced 28-day mortality. The dose of 81 mg/day of aspirin was found to have a less negative effect on patients than 325 mg/day of aspirin because the patients treated with the higher dose of aspirin stayed in the ICU longer.

In terms of original articles, three original studies investigated the effect of BM on respiratory diseases using a pharmacologic network (PN). First, Feng et al. investigated the mechanism by which aloin treats combined allergic rhinitis and asthma syndrome (CARAS). This research employed a PN with molecular docking, molecular dynamics and an experimental approach to investigate the main molecular targets of aloin and its anti-inflammatory effect of this bioactive molecule on ovalbumin-induced CARAS in mice. The results showed that aloin modulated CARAS by inhibiting inflammation and downregulating MAPK signaling-related proteins.

The second study, by Xie et al., investigated the mechanism of action of Qibai Pingfei Capsule (QBPF) using a PN analysis and metabolomics. This study identified that 16 out of 96 metabolites were reversed in the murine COPD model after treatment with QBPF. Additionally, 18 compounds of QBPF, including fumarine and kaempferol exhibited a strong affinity to prostaglandin-endoperoxide synthase 2 (PTGS2). Since PTGS2 is a marker of ferroptosis and is inhibited by QBPF, the results of this study indicate that ferroptosis is involved in COPD pathogenesis.

The original research by Qin et al. studied the treatment of asthmatic mice with Bushenyiqi decoction (BYD) employing a PN. The mechanism of action and pharmacological effects of BYD in asthma remain unclear; however, the authors employed a PN analysis to establish an initial theoretical relationship between BYD and asthma-related genes. Furthermore, the researchers experimentally analyzed the relationship by applying physiological, immunological, and histological assessments in mice with allergic asthma. PN analysis showed that the phosphatidylinositol 3-kinase-RAC-α serine/threonine-protein kinase (PI3K/AKT) signaling pathway is a part of the mechanism of action of BYD for asthma treatment. The results of the experimental study indicated that BYD controls airway inflammation and boosts airway responsiveness. In addition, the results revealed the anti-inflammatory effects of BYD due to quercetin, kaempferol, and luteolin, its bioactive molecules.

On the subject of pulmonary fibrosis, the study by Tsai et al. focused on studying the properties of imperatorin on pulmonary fibroblasts. The authors analyzed the effects of imperatorin on bleomycin-exposed mice and found that zymosan-induced upregulation of connective tissue growth factor (CTGF), α-smooth muscle actin (α-SMA), and collagen protein was diminished. Moreover, imperatorin presented a preventive effect against bleomycin-induced pulmonary fibrosis.

Regarding asthma-chronic obstructive pulmonary disease overlap (ACO), the research of João et al. analyzed plant-derived peptides in a murine model of ACO. This *in vivo* study explored how these peptides affected ACO-related physiological, immunological, and biochemical markers such as airway resistance, cytokine expression, and oxidative stress. Those peptides had similar effects to those of corticosteroids in reversing the ACO responses, including their effect on regulating the hyperresponsiveness, inflammation, remodeling, and oxidative stress markers.

Finally, the contribution by Chernov et al. described the effects of nonpeptide compound TAK-779, an antagonist of CCR5/CXCR3, in the treatment of acute respiratory distress syndrome (ARDS). ARDS has been described as one of the main causes of high mortality in patients with COVID-19. Employing an in-house method to induce unilateral diffuse alveolar damage (DAD) in ICR mice, the authors observed the upregulation of two C–C chemokine receptor 5 (CCR5) ligands, macrophage inflammatory proteins (MIPs) MIP-1α/CCL3 and MIP-1β/CCL4. However, a single administration of TAK-779 showed a reduction of cellular infiltration in lung tissue and decreased inflammatory markers in animals with DAD. These results open the possibility of the use of CCR5 inhibitors to treat virus-induced hyperinflammatory syndromes, including COVID-19 infection.

In summary, this research topic shows the importance of bioactive molecules in the treatment of respiratory diseases, along with the mechanisms of these molecules, and the different types of studies conducted on animals and patients. However, more studies, including clinical trials, are necessary to elucidate and confirm these kinds of properties in the existing literature.
